# Identification of *OSCA* gene family in *Solanum habrochaites* and its function analysis under stress

**DOI:** 10.1186/s12864-022-08675-6

**Published:** 2022-08-01

**Authors:** Shuang Miao, Fengshuo Li, Yang Han, Zhongtong Yao, Zeqian Xu, Xiuling Chen, Jiayin Liu, Yao Zhang, Aoxue Wang

**Affiliations:** 1 College of Horticulture and Landscape Architecture, Northeast Agricultural University, Harbin, 150030 China; 2 College of Life Sciences, Northeast Agricultural University, Harbin, 150030 China; 3 College of Agriculture, Northeast Agricultural University, Harbin, 150030 China; 4 School of Biomedical Engineering, Shanghai Jiao Tong University, Shanghai, 200030 China; 5 College of Sciences, Northeast Agricultural University, Harbin, 150030 China

**Keywords:** *Solanum habrochaites*, *OSCA* gene family, Bioinformatics, *ShOSCA3*

## Abstract

**Background:**

OSCA (hyperosmolality-gated calcium-permeable channel) is a calcium permeable cation channel protein that plays an important role in regulating plant signal transduction. It is involved in sensing changes in extracellular osmotic potential and an increase in Ca^2+^ concentration. *S. habrochaites* is a good genetic material for crop improvement against cold, late blight, planthopper and other diseases. Till date, there is no report on OSCA in *S. habrochaites*. Thus, in this study, we performed a genome-wide screen to identify *OSCA* genes in *S. habrochaites* and characterized their responses to biotic and abiotic stresses.

**Results:**

A total of 11 *ShOSCA* genes distributed on 8 chromosomes were identified. Subcellular localization analysis showed that all members of ShOSCA localized on the plasma membrane and contained multiple stress-related cis acting elements. We observed that genome-wide duplication (WGD) occurred in the genetic evolution of *ShOSCA5 (Solhab04g250600)* and *ShOSCA11 (Solhab12g051500)*. In addition, repeat events play an important role in the expansion of *OSCA* gene family. OSCA gene family of *S. habrochaites* used the time lines of expression studies by qRT-PCR, do indicate OSCAs responded to biotic stress (*Botrytis cinerea*) and abiotic stress (drought, low temperature and abscisic acid (ABA)). Among them, the expression of *ShOSCAs* changed significantly under four stresses. The resistance of silencing *ShOSCA3* plants to the four stresses was reduced.

**Conclusion:**

This study identified the *OSCA* gene family of *S. habrochaites* for the first time and analyzed *ShOSCA3* has stronger resistance to low temperature, ABA and *Botrytis cinerea* stress. This study provides a theoretical basis for clarifying the biological function of OSCA, and lays a foundation for tomato crop improvement.

**Supplementary Information:**

The online version contains supplementary material available at 10.1186/s12864-022-08675-6.

## Background

Tomato (*Solanum lycopersicum*) is an important cash crop and plays an important role in the global vegetable industry [1]. However, environmental stresses like drought, heat, cold and biotic stresses caused by pathogenic microbes and insects are the significant constraints of tomato production, ultimately leading to a severe negative impact on yield [2]. Therefore, more excellent materials and genes need to be identified to cultivate excellent varieties. *S. habrochaites* is a wild relative of cultivated tomato belonging to Solanaceae and Solanum family. It has many excellent agronomic traits, such as cold tolerance, disease resistance and resistance to many other stresses [3]. Therefore, as superior stress resistant materials, *S. habrochaites* often play an essential role in improving the quality characteristics of tomatoes.

As a second messenger, calcium ion (Ca^2+^) participates in regulating the growth and development of plants through sensing external environmental stresses. In living organisms, the core of Ca^2+^ based signal transduction is the concentration of free Ca^2+^ in the cytosol ([Ca^2+^] _cyt_). In non-stimulated or resting cells, the capacity of [Ca^2+^] _cyt_ is in the range of 10^− 7^ M. However, when stimulated, it increased about 10 times to a low level- μM level [4]. One hypothesis is that the mechanism leading to the increase of [Ca^2+^] _cyt_ is the “opening mechanism” [5, 6]. In order to operate Ca^2+^ as an effective signal system, it is necessary to restore [Ca^2+^] _cyt_ to its pre stimulation level, which is realized through the so-called “shutdown mechanism”. The “closure mechanism” covers a series of membrane proteins that move Ca^2+^ along the concentration gradient into or out of the cell’s internal storage areas (such as vacuoles). The result of the switching mechanism is the formation or shaping of Ca^2+^ signal. When plants are directly under biotic stress or abiotic stress, they can respond by forming Ca^2+^ signal [7]. Signals from various stimuli can also activate different Ca^2+^ channels to form Ca^2+^ signals with certain temporal and spatial characteristics, so as to ensure high specificity between stimuli and responses [8]. Known calcium channels in plants include cyclic nucleotide gated channels (CNGCs) [9], two pore channels (TPCS) [10], mechanosensitive channels (MCAS), glutamate receptor channels (GLRS) [11], possibly through Orai channels [12] (at least in plants outside Angiosperm Flora) and recently discovered decreased high osmotic pressure induced Ca^2+^ increased permeability (OSCAs).

Plasma membrane protein OSCA1 is a hypertonic calcium receptor, encoding a protein of 772 amino acids and contains 9 transmembrane domains [13]. OSCA in *A.thalliana* has 15 family members, which are divided into four subfamilies. It contains homologues in other plant species and eukaryotes [14]. Multiple sequence alignment analysis showed that *OSCA* gene family contained a conserved DUF221 domain. DUF221 represents seven transmembrane domains of calcium dependent channels, which are shown in Inter Pro to function as calcium channels in osmotic induction [15]. The detection of *OSCA1* in transgenic *A.thalliana* showed that the gene is expressed in leaves, guard cells and roots [14]. In addition, some studies have found that OSCA responds to osmotic stress and acts as an ion channel for mechanical stimulation. OSCA1.2 can be activated by mechanical force transmitted through the membrane. Its 11 transmembrane helices form a homodimer. The cytoplasmic domain contains an RNA recognition motif and two unique long helices anchored in the lipid bilayer. *OSCA1.2* is very similar to mammalian TMEM16 in structure, but there is no homology in sequence [16–18]. The structure of *OSCA1.1* and *OSCA1.3* were observed by low temperature electron microscope. It is found that the activation of OSCA ion channel will change the conformation of M0 and M6 in the transmembrane helix, straighten M6 and bend M0, resulting in the increase of cross-sectional area of transmembrane region and the opening of ion channel [19]. Therefore, OSCA is a mechanically sensitive Ca^2+^ channel.

Studies have shown that 10 members of *OSCA* gene family in rice respond to drought, salt, ABA and osmotic stress, and the function of four of these in response to osmotic stress has been characterized. During seed water absorption, the functions of 6 *OSCAs* genes were related to osmotic stress, and 3 *OSCAs* were strongly related to circadian rhythm [20]. Twelve *OSCA* gene family members were identified in maize, including *ZmOSCA4.1* gene, which response to drought stress [21]. In *G. hirsutum, G. arboretum* and *G.raimondii*, 35, 21 and 22 gene family members were identified, respectively, including *GhOSCA1.1* a key gene involved in drought and salt tolerance [22]. Recent studies have shown that OSCA responds to plant immune response. In *A.thalliana* PTI immune response, when plasma membrane related cytoplasmic kinase BIK1 is activated, it will be phosphorylated by OSCA1.3. When the channel of OSCA1.3 is opened, Ca^2+^ in cytoplasm increases, forming osmotic potential and regulating stomatal closure of guard cells [23].

No *OSCA* family member in *S. habrochaites* has been characterized till date. Thus, in order to discover the function and genetic evolution of *OSCA* gene family in *S. habrochaites*, we identified *OSCA* genes in *S. habrochaites* by bioinformatics analysis. Further analysis was conducted to identify their molecular characteristics, gene evolution, conserved motif, cis element, protein structure, etc. Their gene expression under biotic (*Botrytis cinerea*) and abiotic stresses (drought, low temperature and ABA) were identified by qRT-PCR, and the function of *ShOSCA3* was validated by VIGS. This study provides an experimental description of the biological function of *OSCA* gene in response to stress and lays a foundation for tomato crop improvement.

## Results

### Identification of *OSCA* gene family members in *S. habrochaites*

**Fig. 1:**
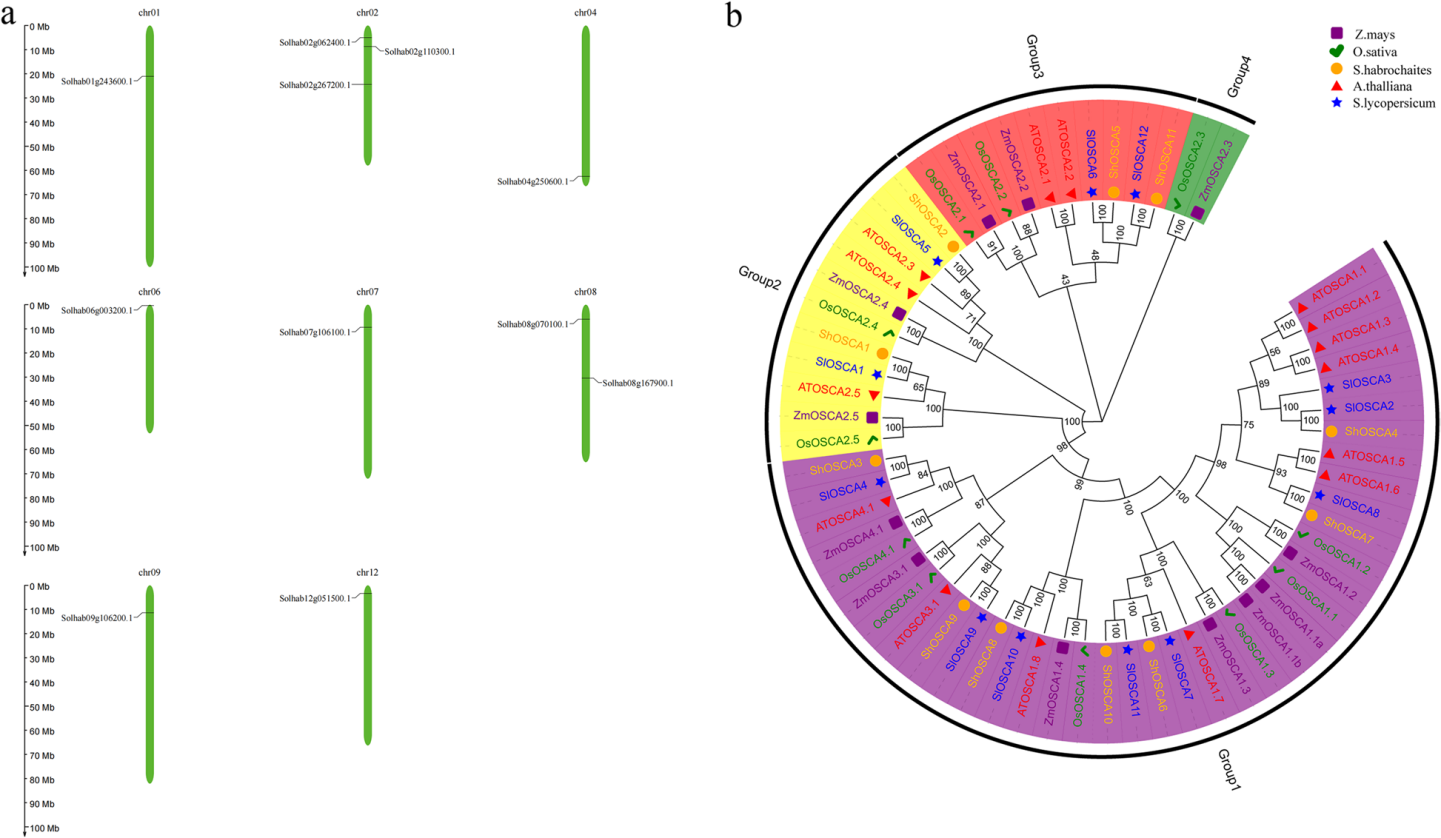
Chromosome locations and phylogenetic analysis of *OSCA* gene family. **a** Chromosome locations of *OSCA* gene family in *Solanum habrochaites*. The chromosome numbers are indicated at the top of each chromosome. Only chromosomes contained *OSCA* genes are represented in this figure. Those genes were named according to their position on the chromosome. **b** Phylogenetic relationships of OSCA protein among *A.thalliana* (*Arabidopsis thaliana L*.), *S.lycopersicum* (*Solanum lycopersicum*), *S.habrochaites* (*Solanum habrochaites*), *O.sativa* (*Oryza sativa* L.), *Z.mays* (*Zea mays* L.). The phylogenetic tree was built by MEGA10 with bootstrap of 1000. Symbols with different colors represent different species

Using the whole genome sequence of *S. habrochaites* and the conservative structure DUF221 of *OSCA* gene family, 11 members of *OSCA* gene family were identified by the bioinformatics method and named according to their position on the chromosome (Table S1). These genes were distributed on 8 chromosomes, respectively (Fig. 1a). Their physical and chemical properties, such as gene length, amino acid length, isoelectric point size and molecular weight, were obtained using the ExPASY website. The gene length ranges from 2142 bp - 2889 bp, and the number of amino acids ranges from 707 aa - 962 aa (Table S1). The isoelectric points of 10 members of the OSCA family of *S. habrochaites* are relatively similar and greater than 7, ranging from 8.58 to 9.39, except that *ShOSCA3* is 6.40. In silico subcellular localization was performed with CELLOv2, and all identified *OSCA* members were located on the plasma membrane, suggesting that *OSCA* genes function in roles related to the plasma membrane.

Evolutionary relationship among OSCA members was identified with phylogenetic trees consisting of a total of 61 protein sequences from *S. habrochaites*, *A.thalliana* (*Arabidopsis thaliana* L.), *S.lycopersicum* (*Solanum lycopersicum*), *O.sativa* (*Oryza sativa* L.), *Z.mays* (*Zea mays* L.). As shown in Fig. 1b, the *OSCA* genes in the five species were classified into four major groups (Groups 1, 2, 3, and 4). Group I contains the most *OSCA* gene members, including 10 members of *A.thalliana* and 7 members of the *S. habrochaites*. Groups 2 and 3 consist of two *OSCA* gene, while group 4 include two *OSCA* genes, *OsOSCA2.3* and *ZmOSCA2.3*.

### Cis acting element analysis

Cis acting elements located upstream of the transcription initiation codon do not code for proteins, but can affect gene expression. 1500 bp upstream of the start codon of *OSCA* genes were obtained with UGENE software, and cis acting elements related to stress contained in them were identified and analyzed with Plant CARE software (Table S2). 82% of the members of *OSCA* gene family in *S. habrochaites* have cis acting element ABRE involving abscisic acid reaction and CGTCA motif involving MeJA reaction. 36% of gene family members have cis acting element TCA involved in salicylic acid reaction. 27% of the gene family members have cis acting element TC-rich which is involved in stress response, cis acting element LTR involved in low temperature response and cis acting element W-box involved in salt response.

### Analysis of conserved motifs and gene structure of *OSCA* gene family in *S. habrochaites*

**Fig. 2:**
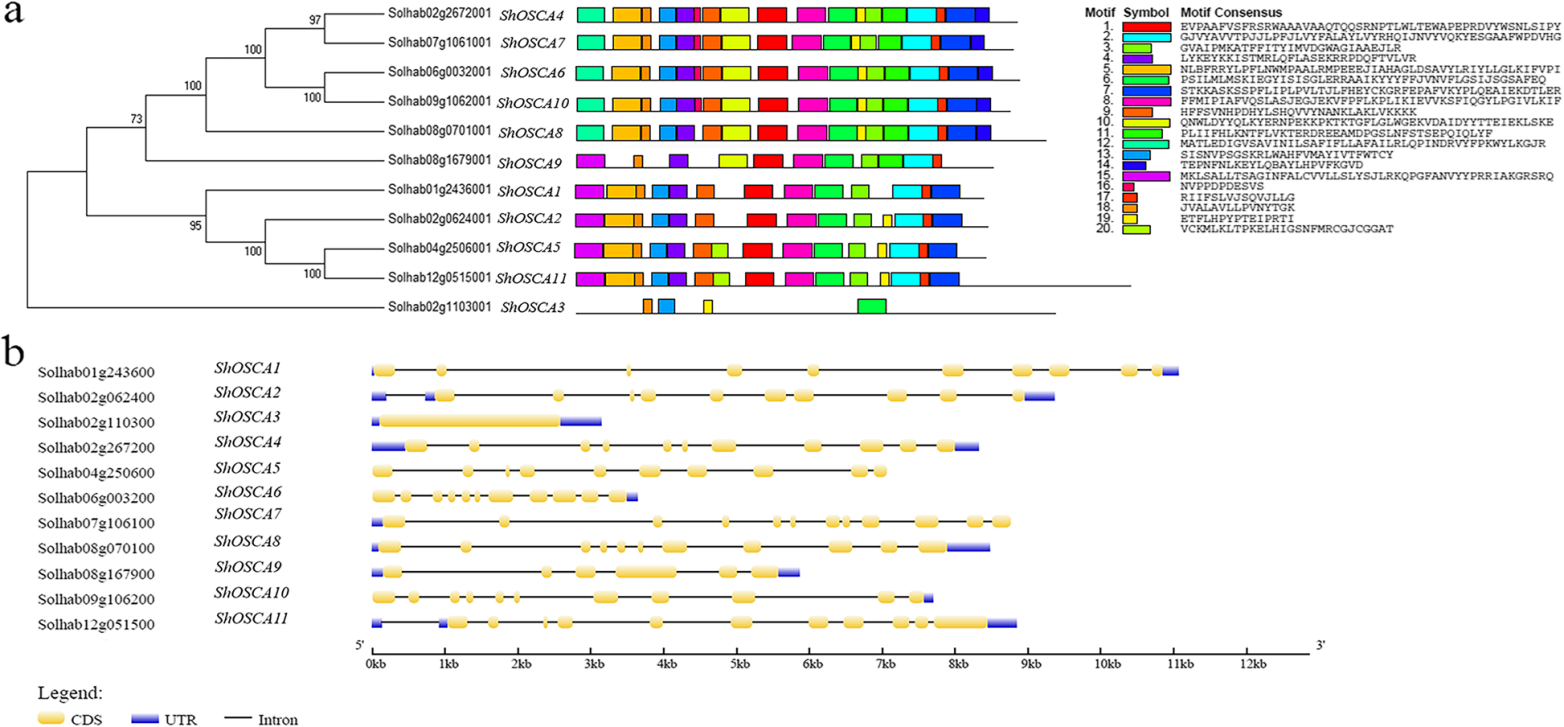
Architecture of conserved protein motifs and gene structure of OSCA gene family. **a** The motif composition of *OSCA* genes. The protein sequences of 11 genes contain 20 conserved motifs, and the same conserved motifs between proteins are represented by the same color; **b** Exon-intron structure of *OSCA* genes. Blue boxes indicate untranslated region (UTR), yellow boxes indicate exons, black lines represent introns

We analyzed *OSCA* gene members in *S. habrochaites* by meme software, and predicted 20 conserved motifs (Fig. 2a). Proteins with similar genetic relationship have similar conserved motifs, indicating that their structure is highly conserved. For example, the conserved motifs and permutation structures of *ShOSCA1, ShOSCA2, ShOSCA5* and *ShOSCA11; ShOSCA4, ShOSCA7, ShOSCA6, ShOSCA10* and *ShOSCA8* are highly similar. GSDS tool was used to analyze the structure of *OSCA* gene in *S. habrochaites*. We found that most members of this family are intron enriched genes. Except for *ShOSCA3*, there are multiple introns in other genes (Fig. 2b).

### Protein structure analysis and function prediction of *OSCA* gene family in *S. habrochaites*

Regions with the same domain usually have similar functions. Some differences in domains among members of *OSCA* genes in *S. habrochaites* may lead to distinct response to stresses. We analyzed the folding mode through analysis of the secondary structure and predicted function through the tertiary structure of the proteins. The results showed that 11 family members contained a large number of α- Spirals and irregular curls, with only a small amount of β-corner (Table S3). The content of α- helix and irregular curl is as high as 94% in these OSCA proteins. In addition to ShOSCA1, ShOSCA3, ShOSCA8 and ShOSCA11, the other 7 members α- helix content reached more than 50%. Their tertiary structure model was constructed with the Phyre2.0 software, revealing that these genes have highly similar protein structure (Fig. S1).

### Synteny analysis and Ka / Ks ratio of *OSCA* gene pairs

**Fig. 3:**
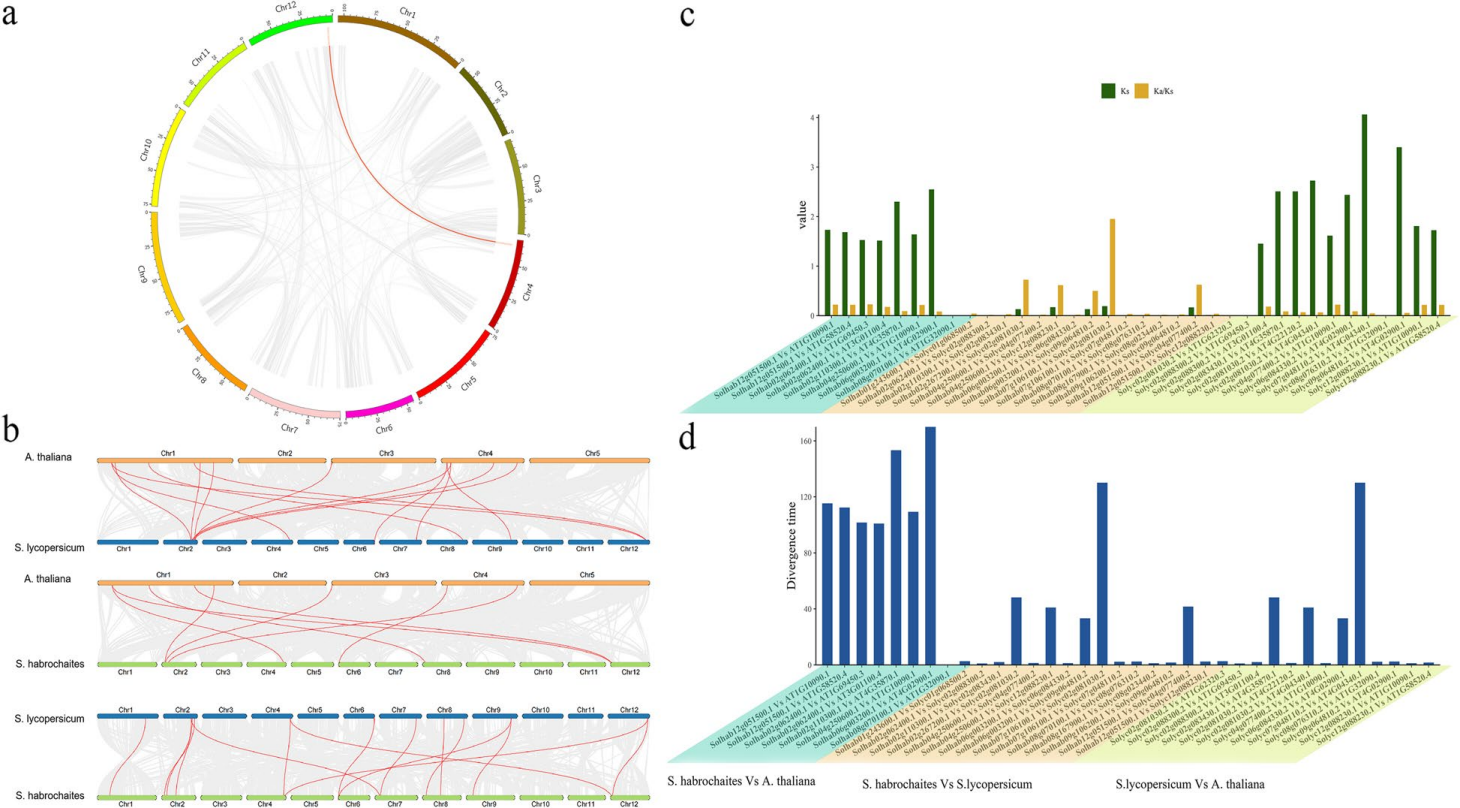
Synteny analyses and Ka/Ks ratio of *OSCA* gene pairs**. a** Syntenic analysis of *OSCA* gene family in *S.habrochaites* genome. The gray line represents the syntenic blocks in the *S.habrochaites* genome, and the red line highlights represents the *OSCA* syntenic gene pair; **b** Syntenic analysis of *OSCA* gene family among genomes. Gray lines in the background represent collinear blocks within the genome of *S. habrochaites* and other plants, while red lines highlight represents the collinearity of *OSCA* gene pairs. The species were *A. thaliana, S. lycopersicum* and *S. habrochaites* respectively; **c** Ks and Ka/Ks values of orthologous *OSCA* gene pairs between any two of the *A. thalliana*, *S.lycopersicum* and *S.habrochaites*; **d** Divergence time estimation of orthologous *OSCA* gene pairs between any two of the *A. thalliana*, *S.lycopersicum* and *S.habrochaites*

Gene replication plays an important role in genome evolution. It can accumulate evolutionary raw materials in the process of replication to promote plant evolution [24]. In particular, genome-wide duplication (WGD) and tandem duplication (TD) have been found to be important for the expansion of gene families [25]. As shown in Fig. 3a, *ShOSCA5 (Solhab04g250600)* and *ShOSCA11 (Solhab12g051500)* had WGD in genetic evolution. To further infer the phylogenetic mechanism among *OSCA* family members, we constructed the comparative syntenic maps of three *OSCA* gene families, namely, the synteny analysis among *A.thalliana*, *S. habrochaites* and *S.lycopersicum* (Fig. 3b). A total of 8 *OSCA* genes in *S.lycopersicum* showed synteny with *A.thalliana*, 6 *OSCA* genes in *S. habrochaites* showed synteny with *A.thalliana*, and 12 *OSCA* genes in *S.lycopersicum* showed synteny with 11 *OSCA* genes in *S. habrochaites*. Some *OSCA* genes were associated with at least two syntentic gene pairs (*OSCA* genes between *S. habrochaites* and *A.thalliana*), such as *ShOSCA2, ShOSCA11* and *SlOSCA3*. In order to better understand the evolutionary constraints acting on *OSCA* gene family, the Ka / Ks ratio of *OSCA* gene pairs and divergence time was calculated (Fig. 3c, d). The Ka / Ks of most direct homologous *OSCA* gene pairs is < 1, indicating that tomato *OSCA* genes may have experienced strong purification and selection pressure in the process of evolution (Fig. 3c). Four pairs, *Solhab08g070100.1* (*ShOSCA8*) versus *AT1G32090.1* (*AtOSCA1.8*), *Solyc02g081030.2* (*SlOSCA3*) versus *AT1G62320.3* (*AtOSCA1.4*), *Solyc02g088300.2* (*SlOSCA5*) versus *AT1G69450.3* (*AtOSCA2.4*), and *Solyc08g076310.2* (*SlOSCA10*) versus *AT1G32090.1* (*AtOSCA1.8*) Ks values could not be calculated, indicating that there might be more sequence divergence between these genes.

### Expression analysis of OSCA gene family in *S. habrochaites* under stress

**Fig. 4:**
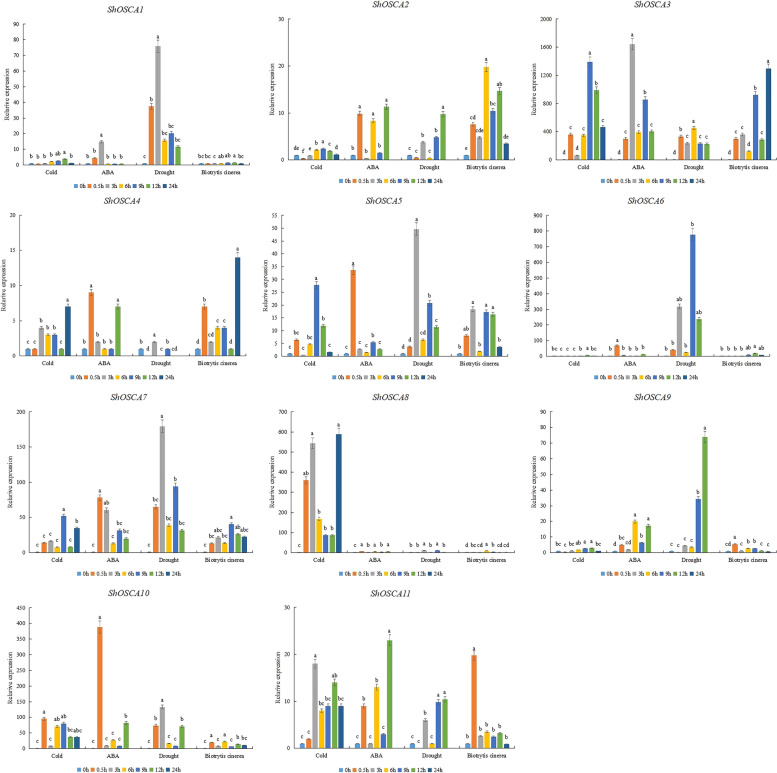
Expression analysis of *OSCA* genes under 4 °C, ABA, drought and *Botrytis cinerea* treatment. The small letters mean significant differences at *P* < 0.05 (Tukey)

We hypothesized that the expression of *ShOSCAs* genes might respond to a series of plant stress because bioinformatic analysis revealed the presence of stress-associated cis-acting elements in their promoter. Therefore, to further analyze the function of *OSCA*, the gene expression under *Botrytis cinerea* and drought, low temperature and ABA stresses was evaluated by qRT-PCR. We set the expression of 0 h as 1 and compared the expression at other time points. As shown in Fig. 4, under drought stress, the expression levels of *ShOSCA1, ShOSCA3, ShOSCA6* and *ShOSCA9* were the highest at 0.5, 3, 9 and 12 h after treatment. Among them, the expression of *ShOSCA8* was significantly up-regulated at 3 and 12 h in response to low temperature stress. The expression of *ShOSCA10* reached the maximum at 0.5 h after ABA treatment. Under *Botrytis cinerea* stress, the gene expression of *ShOSCA3* was significantly up-regulated at 9 and 12 h. As shown in Fig. 4, the expression of *ShOSCA3* was significantly up-regulated under four stress treatments, indicating that *ShOSCA3* responds to *Botrytis cinerea* drought, low temperature and ABA stresses.

### Functional characterization of ShOSCA3

We used functional analysis by VIGS to characterize the function of *ShOSCA3*. Firstly, we constructed VIGS vector *PTRV2-ShOSCA3* and transferred the silencing vector *PTRV2-PDS* (phytoene dehydrogenase), an indicator gene of the VIGS system into *S. habrochaites* by *Agrobacterium* infection. After 17 days of tomato inoculation, *ShOSCA3* silenced plants began to turn white from the veins in new leaves (Fig. S2, a, b, c), and the silencing efficiency of *ShOSCA3* was confirmed by qRT-PCR (Fig. S2, d). When the plant is damaged by stress, a large amount of ROS is produced, which will poison the cells, causing membrane damage and cell death. Therefore, the oxidative damage degree of *ShOSCA3* silenced plants and control plants was evaluated by measuring the contents of superoxide dismutase (SOD), peroxidase (POD), polyphenol oxidase (PPO) and ascorbic acid peroxidase (APX) under *Botrytis cineria*, low temperature, drought and ABA stress.

**Fig. 5:**
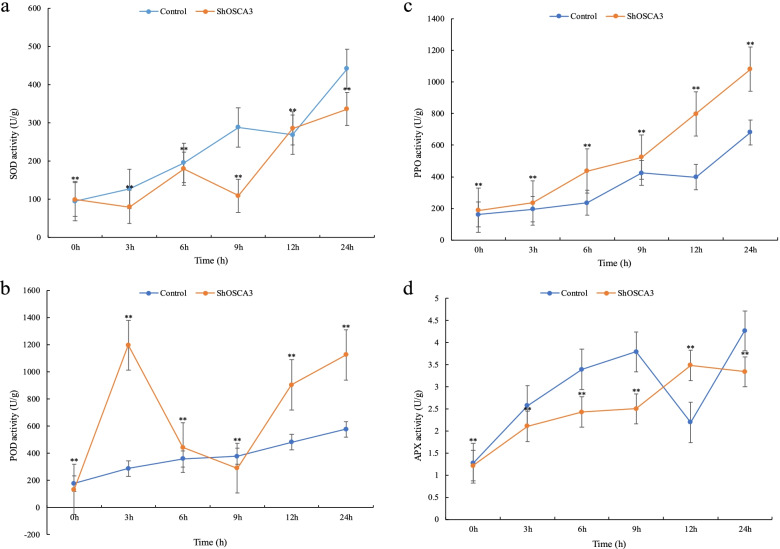
Effects of *Botrytis cinerea* stress on SOD, POD, PPO and APX of *ShOSCA3* silencing group. Error bars represent the standard deviation of three independent biological replicates. One-way ANOVA test was used for significance analysis. “**” represents a significant difference between the data point and control (*p* < 0.01, LSD)

Under *Botrytis cinerea* stress, as shown in Fig. 5, the SOD content of control plants and *ShOSCA3* silenced plants showed a gradually increasing trend, but the SOD content of control plants was higher than that of *ShOSCA3* silenced plants. The POD activity of *ShOSCA3* silenced plants was higher than that of control plants. The changing trend of PPO content was gradually increasing, but the PPO content of *ShOSCA3* silenced plants was higher than that of control. The study of APX content found that the changing trend of *ShOSCA3* silenced plants was consistent with that of the control plants, but at 12 h, the changing trend was the opposite, and the APX activity of the control plants was higher.

**Fig. 6:**
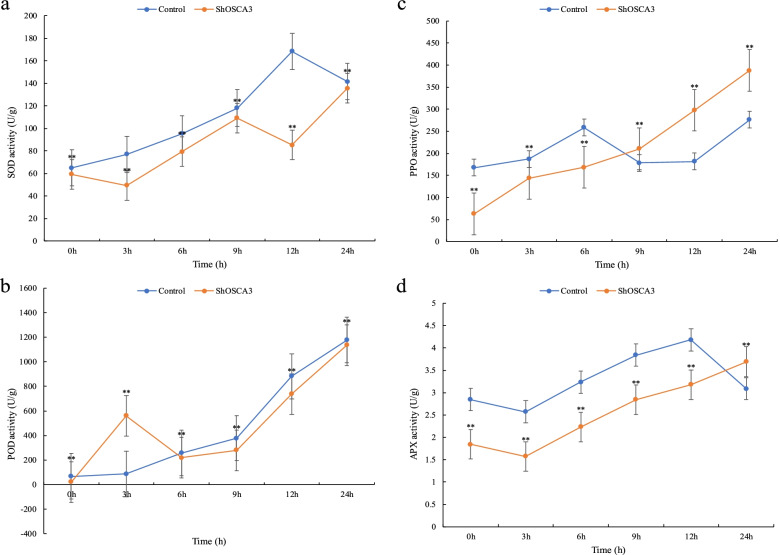
Effects of cold stress on SOD, POD, PPO and APX of *ShOSCA3* silencing group. Error bars represent the standard deviation of three independent biological replicates. One-way ANOVA test was used for significance analysis. “**” represents a significant difference between the data point and control (*p* < 0.01, LSD)

After 3 hours of exposure to low temperature, the expression of APX increased sharply. After 12 hours, the content of APX in the control group decreased and was lower than that of *ShOSCA3* silenced plants. The results showed that the resistance of *ShOSCA3* silenced plants under cold stress was weaker than that of the control plants. The amount of superoxide dismutase (SOD) and peroxidase (POD) in *ShOSCA3* silenced plants were lower than those in control plants. After 9 hours of cold stress, the PPO content of *ShOSCA3* silenced plants was higher than that of control plants, but the overall trend of PPO content was gradually increased (Fig. 6).

**Fig. 7:**
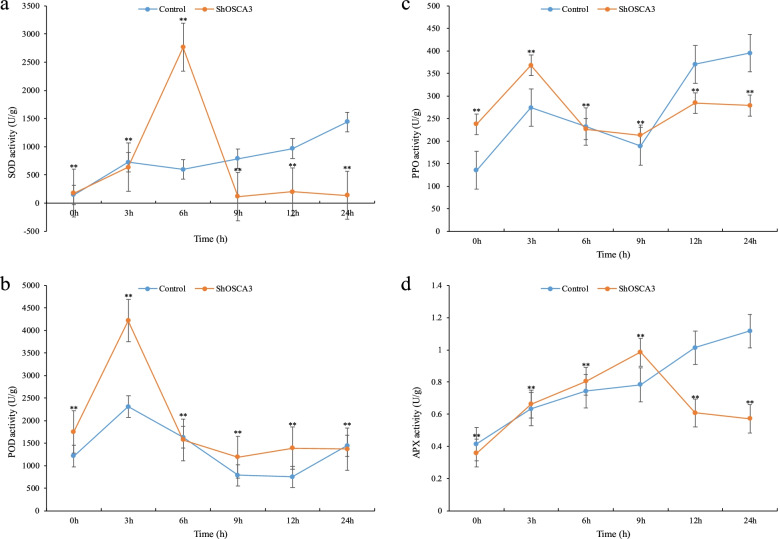
Effects of drought stress on SOD, POD, PPO and APX of *ShOSCA3* silencing group. Error bars represent the standard deviation of three independent biological replicates. One-way ANOVA test was used for significance analysis. “**” represents a significant difference between the data point and control (*p* < 0.01, LSD)

Under drought stress, the SOD content of *ShOSCA3* silenced plants increased sharply at 6 h, and the content was lower than that of the POD in *ShOSCA3* silenced plants, Control plants increased sharply at 3 h, but the overall trend of POD content in *ShOSCA3* silenced plants was higher than that of control plants. The change in PPO content showed a similar trend between the control group and the silenced group. The trend of APX content was the same before 9 h, and then the APX content of *ShOSCA3* silenced plants was slightly lower than that of the control (Fig. 7).

**Fig. 8:**
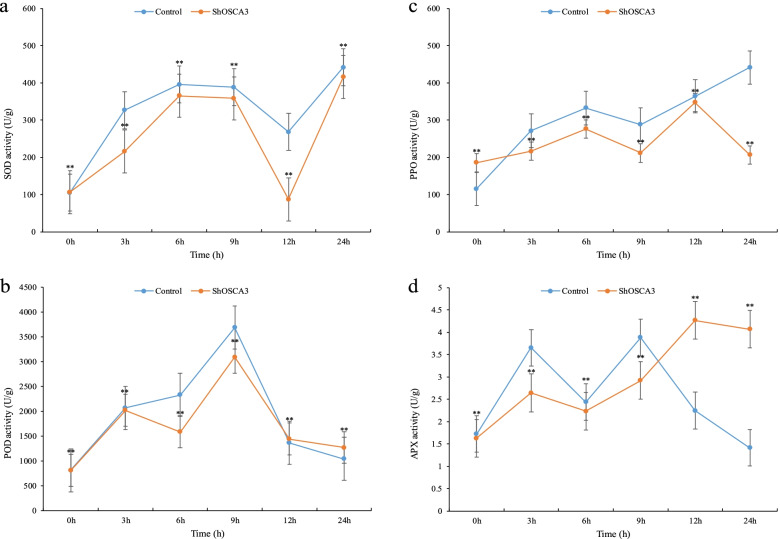
Effects of ABA stress on SOD, POD, PPO and APX of *ShOSCA3* silenced group. Error bars represent the standard deviation of three independent biological replicates. One-way ANOVA test was used for significance analysis. “**” represents a significant difference between the data point and control (*p* < 0.01, LSD)

As shown in Fig. 8, under ABA stress, the overall trend of SOD content in silenced plants and control plants was the same, and it showed a downward trend at 12 h, but the control group was higher than the silenced group on the whole. The changing trend of POD content in the control and the silencing group was similar, and the POD content increased at 3 h and 6 h. The fluctuation trend of PPO in control group and the transgenic silenced group was similar, but the content of PPO in control group was higher than that in the silencing group. The changing trend of APX content in control and silencing group was similar before 9 h, and the APX content in the silencing group increased after 9 h (Fig. 8).

## Discussion

*S. habrochaites* is a good genetic material for crop improvement against cold, late blight, planthopper and other diseases [26, 27]. *S. habrochaites* can be genetically crossed with cultivated tomato only when used as male parent, but the seed setting rate is low [28]. Therefore, it is difficult to transfer the excellent characteristics of *S. habrochaites* to cultivated varieties. In this study, *OSCA* genes were identified in *S. habrochaites* and the role under stress was further evaluated, thus, laying a foundation for the genetic improvement of cultivated tomato.

In this study, a total of 11 *OSCA* genes were identified in *S. habrochaites*. Compared with cultivated tomato, *S. habrochaites* has lost one OSCA gene during evolution, and four in comparison with *A.thalliana*, but has the same number of *OSCA* genes as in rice [20]. However, according to the phylogenetic tree constructed, the four species are divided into four branches. This shows that rice, *S. habrochaites*, *S.lycopersicum* and *A.thalliana* have experienced relatively conserved evolution. The occurrence of gene replication events will lead to the contraction and expansion of the gene family and provide complexity for gene function [29]. The collinearity results of *A. thaliana*, *S. lycopersicum* and *S. habrochaites* showed that the amount of replication events occurred in the evolution of *OSCA* gene.

Conserved genes are more likely to produce functional and lasting duplication. These genes are often involved in signal transduction and regulation, which contributes to their long-term viability in the eukaryotic genome [30, 31]. Such proteins that encode multiple domains and have multiple cis regulatory regions will be preferentially expressed [32]. This study revealed that the promoter regions of *OSCA* gene family contain cis acting elements associated with many stresses, indicating that they have obtained more diverse functions in gene evolution. In addition to *ShOSCA8* and *ShOSCA9*, members of the *OSCA* gene family of *S. habrochaites* contain ABRE cis acting elements, which can reverse activate and produce a large amount of ABA, involved in ABA signal transduction pathway [33, 34]. In addition, *OSCA* gene family of *S. habrochaites* also contains cis acting elements CGTCA motif and TGACG motif involved in jasmonic acid signal transduction pathway, and cis acting element TCA of salicylic acid signal transduction pathway. Jasmonic acid and salicylic acid are indispensable in plant disease resistance, but they have a certain antagonistic effect [35, 36]. The *OSCA* gene family of *S. habrochaites* contains these cis acting elements, indicating that these genes are likely involved in disease resistance. In addition, *ShOSCA4* and *ShOSCA9* contain homeopathic elements circadian related to circadian rhythm, while in rice, *OsOSCA1.2, OsOSCA2.1* and *OsOSCA2.2* also have the same function [20]. The result further shows that *OSCA* gene is conservative in gene function in the process of evolution, even if gene replication events continue to occur.

RNA seq data on the response of *S. habrochaites* to various stresses need to be improved. RNA seq data combined with gene expression pattern analysis can help understand gene function better. According to the expression pattern of *OSCA* gene in *S. habrochaites* under low temperature, drought, ABA and *Botrytis cinerea* stress, it was found that the expression of *ShOSCA3* changed significantly under various stresses. The gene structure was analyzed and it was found that the gene had no intron. Genes with few or no introns are thought to be rapidly expressed in plants [37]. The longer the intron, the longer the gene transcription time, and the faster the genes with fewer introns respond to biotic and abiotic stresses. For example, some stress-related gene families, such as Hsp20 family [38], leucine rich repeat family [39] and GRF family [40], also contain a few introns. Therefore, without intron, it may gain the advantage of time in the process of transcription and can quickly respond to all kinds of stress. In addition, corn *ZmOSCA4.1* and *ShOSCA3* are in the same evolutionary branch, and there is no intron in their gene structure. However, under drought stress, the change of *ZmOSCA4.1* is the most significant. The expression of *ShOSCA3* also changed significantly under drought stress, which further indicates the conservation of gene function in the process of evolution. In addition, *ZmOSCA4* response under other stress still needs to be evaluated.

In order to further analyze the gene function of *ShOSCA3,* the gene was silenced by VIGS technology. The control plants and *ShOSCA3* silenced plants were subjected to low temperature, drought, ABA and *Botrytis cinerea* stress, and the stress-related physiological indexes were detected. After plants were subjected to biotic and abiotic stress, the balance of reactive oxygen species in plants is distorted and ROS is accumulated in large quantities, resulting in toxic effects on cells [41]. Therefore, detecting the main enzyme activity of the active oxygen scavenging mechanism of *ShOSCA3* silenced plant is one of the main methods to detect the damage of plants under stress and analyze the function of the silenced gene. ABA is one of the important endogenous hormones in plants and the critical endogenous messenger in response to a variety of stress [42]. We discovered that the PPO activity of *ShOSCA3* silenced plants was lower than that of the control, indicating that the PPO related response mechanism was inhibited under ABA stress [43]. Under ABA treatment, the activities of SOD, POD and APX in control were higher than those in *ShOSCA3* silenced plants, indicating that *ShOSCA3* silenced plants had the ability to resist the high concentration of ABA stress. Plant disease resistance is closely related to the accumulation of phenylpropane metabolites, the expression of pathogen related protein (PRP), pentose phosphate pathway, glyoxylate cycle and other processes [44]. The key enzymes in disease resistance metabolism include phenylalanine ammonia lyase (PAL), peroxide (POD), polyphenol oxidase (PPO) and so on. The change of its activity is one of the effective indexes to reflect the strength of plant disease resistance. Under the infection of *Botrytis cinerea*, the PPO enzyme activity in *ShOSCA3* silenced plants was higher than that of control plants, and the PPO enzyme activity was also higher than in control plants. This indicates that the plant is more susceptible to disease after silencing *ShOSCA3*, resulting in the increase of disease resistance related enzyme activity, but it does not affect the response of plant disease resistance, which indicates that *ShOSCA3* respond to early plant immune response. We will further understand how *ShOSCA3* participates in various stresses and its effect on the change of cytoplasmic Ca^2+^ concentration.

## Conclusion

In this study, 11 *OSCA* gene family members were identified in *S. habrochaites* and localized in the cytoplasmic membrane. Phylogenetic results divided these *OSCA* genes into 4 groups. Structural analysis showed that *ShOSCAs* is a gene family rich in introns. *OSCA* gene was subjected to biotic stress (*Botrytis cinerea*) and abiotic stress (drought, ABA, cold). qRT-PCR results showed that *ShOSCA3* responded strongly to multiple stresses. *ShOSCA3*-silenced plants were subjected to stress treatment and assayed for stress marker enzyme activity, which indicated that *ShOSCA3* was more resistant to low temperature, ABA and botrytis stress.

## Materials and methods

### Plant materials and treatments

*S. habrochaites* (LA1777) was provided by tomato genetics research center (TGRC, https://tgrc.ucdavis.edu/). The seeds were disinfected with 5% sodium hypochlorite for 15 min, then washed with 75% alcohol once, followed by three times wash with sterile water. Finally, they were placed on wet filter paper and cultured with soil and vermiculite in a ratio 3:1 matrix. The illumination time was 16 h and 8 h night time at 25 °C. Stress treatment was carried out at the five leaves stage. *Botrytis cinerea* infection: *B. cinerea* spores was diluted with an appropriate amount of sterile water to make the concentration of spore suspension 10^7^ CFU/mL. Then, the bacterial solution was sprayed on the tomato plants, and cultured in a light incubator with 90% humidity and 20–25 °C, while the control plants were sprayed with water. The sampling time points were 0, 0.5, 3, 6, 9, 12 and 24 h after *B. cinerea* treatment. Drought stress: the plants were cultured in 20% PEG6000 (1/2 Hoagland nutrient solution) [45], and the sampling time points were 0, 0.5, 3, 6, 9 and 12 h respectively. ABA treatment: 150 μM / mL ABA was added to 1/2 Hoagland nutrient solution. The sampling time points were 0, 0.5, 3, 6, 9 and 12 h, respectively. Cold stress: the plants were placed in a 4 °C incubator (PR205740RCN, Thermo Scientific, USA) for 16 hours of light and 8 hours of darkness. The sampling time points were 0, 0.5, 3, 6, 9, 12 and 24 h, respectively. All treated tissue samples were immediately frozen in liquid nitrogen and stored at − 80 °C. Three plants with the same growth were taken from each treatment for three biological replicates. Three technical replicates were performed for each treatment.

### Bioinformatics analysis of *OSCA* gene family in *S. habrochaites*

The whole-genome sequence of *S. habrochaites* was assessed from the website http://www.solomics.neau.edu.cn. According to the characteristics of *OSCA* gene family with the conserved structure (DUF221, also known as RSN1_7TM, PfamID: PF02714), which was the screening condition, the hidden Markov model (HMM) file corresponding to DUF221 was downloaded from the Pfam protein family database (http://pfam.sanger.ac.uk/). Then the protein sequence data of *S. habrochaites* were searched by using the default parameters of HMMER v3.0 [46]. The transcripts were input into CDD and Pfam for to detect the conserved DUF221 domain. Finally, the sequence with the complete DUF221 domain was retained and named according to its position on the chromosome. The molecular weight, isoelectric point and subcellular location of proteins in the identified *OSCA* gene family were calculated using the ExPASY website (http://web.expasy.org/protparam/).

### Phylogenetic relationships analysis and chromosomal localization

The *OSCA* gene sequences of maize (*Zea mays* L.) was downloaded from the MaizeGDB database (https://www.maizegdb.org/), and the *OSCA* gene sequences of rice (*Oryza sativa* L. ssp. Japonica). *A.thalliana* was downloaded from the NCBI Conserved Domain Database (www.ncbi.nlm.nih.gov/Structure/cdd/wrpsb.cgi). For *S.lycopersicum,* they were retrieved from the Sol Genomic Network (https://solgenomics.net/). We used MEGA7.0 software (neighbor joining adjacency method) to draw the system evolution tree, and the parameters were as follows: Poisson model; Pairwise deletion; the bootstrap method is set to 1000. Based on the nucleotide sequence of the *OSCA* gene family members obtained above, Mapchart 2.32 software was used to construct the chromosome mapping.

### Genic structure and protein structural analysis

The gene sequence 1500 bp upstream of the start codon of *OSCA* gene family was obtained from the genome sequence of *S. habrochaites* by UGENE software. The cis acting elements located in the promoters of each members were identified by obtaining the genomic sequence upstream of each gene and analyzed in the Plant CARE software (http://bioinformatics.psb.ugent.be/webtools/plantcare/html/). Using online program gene structure display server (GSDS: http://gsds.cbi.pku.edu.cn/), the predicted coding sequence of *S. habrochaites* was compared with the corresponding full-length sequence, and the exon-intron structure diagram was drawn [47]. The secondary structure of the identified protein was simulated with SOPMA software, and then Phyre2.0 software (http://www.sbg.bio.ic.ac.uk/ ≈ phyre /) was.

### Synteny analyses

The gene replication events among *OSCA* members in *A.thalliana*, *S. habrochaites* and *S.lycopersicum* were analyzed by multicollinearity scanning Toolkit (MCScanX), using the default parameters [48, 49]. KaKs_Calculator 2.0 was used to calculate the synonymous (Ks) and non-synonymous (Ka) substitutions for each repeat [50]. The divergence time was calculated by the formula T = Ks/r, where “r “was the divergence rate of the plant nuclear gene. For dicotyledonous plants, the r was taken to be 1.5× 10^−8^ synonymous substitutions per site per year.

### Quantitative reverse transcription polymerase chain reaction (qRT-PCR) analysis

Total RNA was extracted from plant leaves cultured at 25 °C for 3 weeks with Trizol (Invitrogen, USA) reagent, and qRT PCR was performed with Chamq SYBR qPCR Master Mix (without Rox) (Vazyme, Nanjing, China). Sequence of primers used are listed in Table S1. Each reaction contains 10 μL Chamq SYBR qPCR Master Mix (Without Rox) and 0.4 μL forward primer (10 μM), 0.4 μL reverse primer (10 μM) and cDNA samples diluted 10 times with 1.0 μL. The reaction process was as follows: reaction conditions: 95 °C / 30 s; 95 °C / 10 s, 60 °C / 30 s, 40 cycles in total; Then 95 °C / 15 s, 60 °C / 60 s, 95 °C / 15 s. The relative expression level was calculated using the 2^-ΔΔct^ method [51]. Each reaction consisted of three technical replicates, and the housekeeping gene, *Actin,* was used as the internal reference. Data represent means of three replicates ± standard deviation (SD). Analysis was performed using the Data Processing System, and Turkeys multiple range test were conducted to determine significant differences. *P* < 0.05 was considered to indicate statistical significance.

### Virus-induced gene silencing (VIGS)

The constructed *PTRV2-ShOSCA3, PTRV1* and *PTRV2 -PDS* vectors were transformed into Agrobacterium strain *EHA105* using the *Agrobacterium tumefaciens* transformation method. Cell culture containing the appropriate plasmid was injected into the tomato seedlings through the leaves with a syringe. Infected tomatoes were cultured overnight at 22 °C in the dark, and then cultured in a greenhouse at 21 °C, 30% humidity and 16 h / 8 h photoperiod. After the positive control plants turned completely white, the leave samples were obtained from the test and the control group. The efficiency of gene silencing was evaluated with qRT-PCR.

### Enzyme activity detection of SOD, POD, PPO and APX

*ShOSCA3* silenced plants were exposed to low temperature (4 °C), drought (20% PEG6000) ABA (150 μM / mL) and Botrytis cinerea treatment. Samples were taken at 0, 3, 6, 9, 12 and 24 h, and enzyme activities were determined using Kit (Solarbio, China). The absorbance values for SOD, POD, PPO and APX were measured at 560 nm, 470 nm, 410 nm and 290 nm, respectively. One-way ANOVA test was used for significance analysis. “**” represents a significant difference between the data point and control (*p* < 0.01, LSD). All data were expressed as the mean ± SD (stander deviation) after normalization of the three independent experiments.

## Supplementary Information

**Additional file 1: Table S1**. *OSCAs* gene family information of *S. habrochaites.* **Table S2.** Analysis of cis acting elements of *OSCA* gene family in *S. habrochaites.* **Table S3**. Protein structure of *OSCA* gene family in *S. habrochaites.* **Table S4**. Primers used for qRT-PCR. **Fig. S1**. Protein tertiary structure of *OSCA* gene family in *Solanum habrochaites.* **Fig. S2**. *ShOSCA3* gene expression in silenced plants. a, b, c, plant phenotypes of *pTRV2-PDS*, *pTRV2*, *pTRV2-ShOSCA3* at 17 days of inoculation of *Solanum habrochaites*; d, Silencing of *ShOSCA3* confirmed by qRT-PCR.

## Data Availability

The datasets used and/or analysed during the current study available from the corresponding author on reasonable request.
