# Supplementary material for: Identification of OSCA gene family in Solanum habrochaites and its function analysis under stress
Source: BMC Genomics. 2022 Aug 1;23:547. doi: 10.1186/s12864-022-08675-6 (PMC9341080; doi:10.1186/s12864-022-08675-6)
Supplement: Supplementary file 1 — Additional file 1: Table S1. OSCAs gene family information of S. habrochaites. Table S2. Analysis of cis acting elements of OSCA gene family in S. habrochaites. Table S3. Protein structure of OSCA gene family in S. habrochaites. Table S4. Primers used for qRT-PCR. Fig. S1. Protein tertiary structure of OSCA gene family in Solanum habrochaites. Fig. S2. ShOSCA3 gene expression in silenced plants. a, b, c, plant phenotypes of pTRV2-PDS, pTRV2, pTRV2-ShOSCA3 at 17 days of inoculation of Solanum habrochaites; d, Silencing of ShOSCA3 confirmed by qRT-PCR. [file 12864_2022_8675_MOESM1_ESM.docx]

**Supplementary Materials:**

Table S1 *OSCAs* gene family information of *S. habrochaites*

| Gene name | Sequence accession | Gene Length（bp） | Protein length  (aa) | PI | Mv/ Da（ku） | Subcellular localization |
| --- | --- | --- | --- | --- | --- | --- |
| *ShOSCA1* | *Solhab01g243600* | 2124 | 707 | 8.87 | 80929.1 | Plasma Membrane |
| *ShOSCA2* | *Solhab02g062400* | 2151 | 716 | 8.87 | 81100.8 | Plasma Membrane |
| *ShOSCA3* | *Solhab02g110300* | 2496 | 831 | 6.40 | 94048.0 | Plasma Membrane |
| *ShOSCA4* | *Solhab02g267200* | 2298 | 765 | 9.39 | 87602.4 | Plasma Membrane |
| *ShOSCA5* | *Solhab04g250600* | 2142 | 713 | 9.43 | 80882.9 | Plasma Membrane |
| *ShOSCA6* | *Solhab06g003200* | 2301 | 766 | 9.14 | 88058.9 | Plasma Membrane |
| *ShOSCA7* | *Solhab07g106100* | 2274 | 757 | 8.58 | 86679.9 | Plasma Membrane |
| *ShOSCA8* | *Solhab08g070100* | 2448 | 815 | 9.19 | 93965.6 | Plasma Membrane |
| *ShOSCA9* | *Solhab08g167900* | 2172 | 723 | 9.29 | 81976.2 | Plasma Membrane |
| *ShOSCA10* | *Solhab09g106200* | 2256 | 751 | 9.29 | 85931.7 | Plasma Membrane |
| *ShOSCA11* | *Solhab12g051500* | 2889 | 962 | 9.33 | 108456.0 | Plasma Membrane |

Table S2 Analysis of cis acting elements of *OSCA* gene family in *S. habrochaites*

| No. | Name | Function | 1 | 2 | 3 | 4 | 5 | 6 | 7 | 8 | 9 | 10 | 11 |
| --- | --- | --- | --- | --- | --- | --- | --- | --- | --- | --- | --- | --- | --- |
| 1 | TC-rich | defense and stress response |  | √ |  |  |  |  |  |  | √ | √ |  |
| 2 | ABRE | abscisic acid reaction | √ | √ | √ | √ | √ | √ | √ |  |  | √ | √ |
| 3 | ARE | Involving anaerobic induction | √ | √ | √ |  | √ | √ |  | √ |  | √ |  |
| 4 | CGTCA-motif | Jasmonic acid reaction |  | √ |  | √ | √ | √ | √ | √ | √ |  | √ |
| 5 | LTR | Cold response |  |  |  |  | √ | √ |  | √ |  |  |  |
| 6 | P-box | Gibberellin reaction |  |  |  |  |  |  | √ |  |  | √ |  |
| 7 | TATC-box | Involving gibberellin reaction |  |  |  | √ |  |  |  |  |  |  |  |
| 8 | TCA-element | Involving salicylic acid reaction | √ |  |  | √ |  | √ |  | √ |  |  |  |
| 9 | TGA-element | Auxin reaction |  |  |  |  |  | √ |  |  |  | √ |  |
| 10 | TGACG-motif | Jasmonic acid reaction |  | √ |  | √ | √ | √ | √ | √ | √ |  | √ |
| 11 | W-box | Involving salt, ABA |  |  | √ |  |  |  |  |  |  | √ | √ |
| 12 | circadian | Involving circadian regulation |  |  |  | √ |  |  |  |  | √ |  |  |

Note:1-11 indicates *ShOSCA1-11.*

Table S3 Protein structure of *OSCA* gene family in *S. habrochaites*

| protein | α-helix% | β-corner% | Randomcoil% |
| --- | --- | --- | --- |
| *ShOSCA1* | 38.25% | 2.49% | 45.01% |
| *ShOSCA2* | 54.75% | 1.26% | 30.45% |
| *ShOSCA3* | 48.98% | 5.54% | 31.17% |
| *ShOSCA4* | 52.81% | 1.83% | 32.94% |
| *ShOSCA5* | 51.19% | 2.38% | 32.82% |
| *ShOSCA6* | 50.52% | 2.09% | 33.55% |
| *ShOSCA7* | 52.84% | 1.98% | 32.23% |
| *ShOSCA8* | 48.34% | 3.56% | 32.88% |
| *ShOSCA9* | 54.91% | 1.66% | 31.81% |
| *ShOSCA10* | 51.53% | 2.93% | 32.09% |
| *ShOSCA11* | 40.85% | 2.49% | 43.14% |

Table S4 Primers used for qRT-PCR

| Prime name | Upstream primer (5’-3’) | Downstream primer (3’-5’) |
| --- | --- | --- |
| *ShOSCA1* | GCCGACTCTACTTAGGCCAC | CGGAAGCAATCGCCGTAAAG |
| *ShOSCA2* | GCAGCCAGGAAATGCAGAAG | TGCGCATGAAAACAACAGCA |
| *ShOSCA3* | AGATGTGGCTGAACTGGGTG | TAGCTCAGCCCTTTGCTCAC |
| *ShOSCA4* | GTACAAAACGCGCTGCTCAA | TGGTGAAAGCTGGCTCGTAG |
| *ShOSCA5* | ACTGATGCAGGGAAGGTGTG | GCATGCTGAACGGTGTTGTT |
| *ShOSCA6* | TGAGCACCAAAGAGGCTTCC | ACTGTATCCTTCACCACCGC |
| *ShOSCA7* | AGGTCGTTTTGAGCCAGCTT | GTGAAGGTTCCAGATCTCCGT |
| *ShOSCA8* | TGCTCTGCTTAGACTTCAGCC | CCTTGGAGCACTCCTCTTCC |
| *ShOSCA9* | GAGCATCGAGCATGATCCCA | GATAGTTCCAGCCCGTAGCC |
| *ShOSCA10* | TGCAAGGGCCGATATGAGTC | CTGACGGTCTGTCCAACTCC |
| *ShOSCA11* | TGTATCAACAACTCACATCAGCTT | GGAGCAGCTTTACCCAGTGA |
| *Actin* | TGTCCTATTTACGAGGGTTATGC | CAGTTAAATCACGACCAGCAAGAT |

*
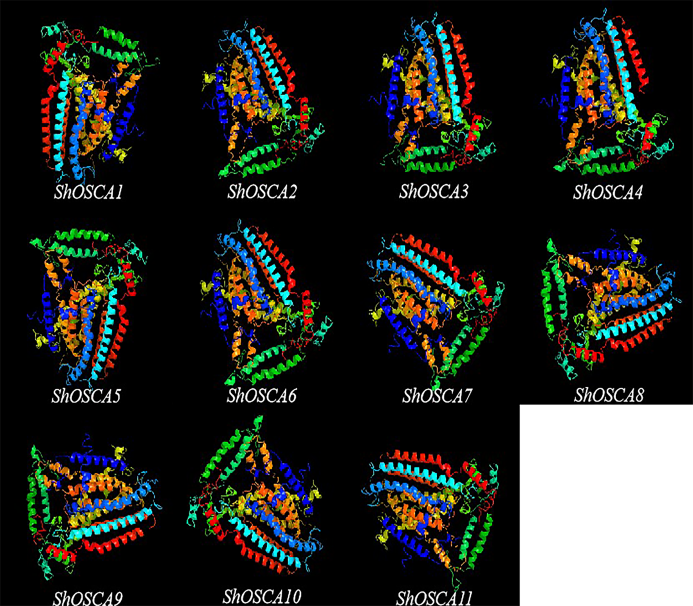
*

Fig. S1 Protein tertiary structure of *OSCA* gene family in *S. habrochaites.*


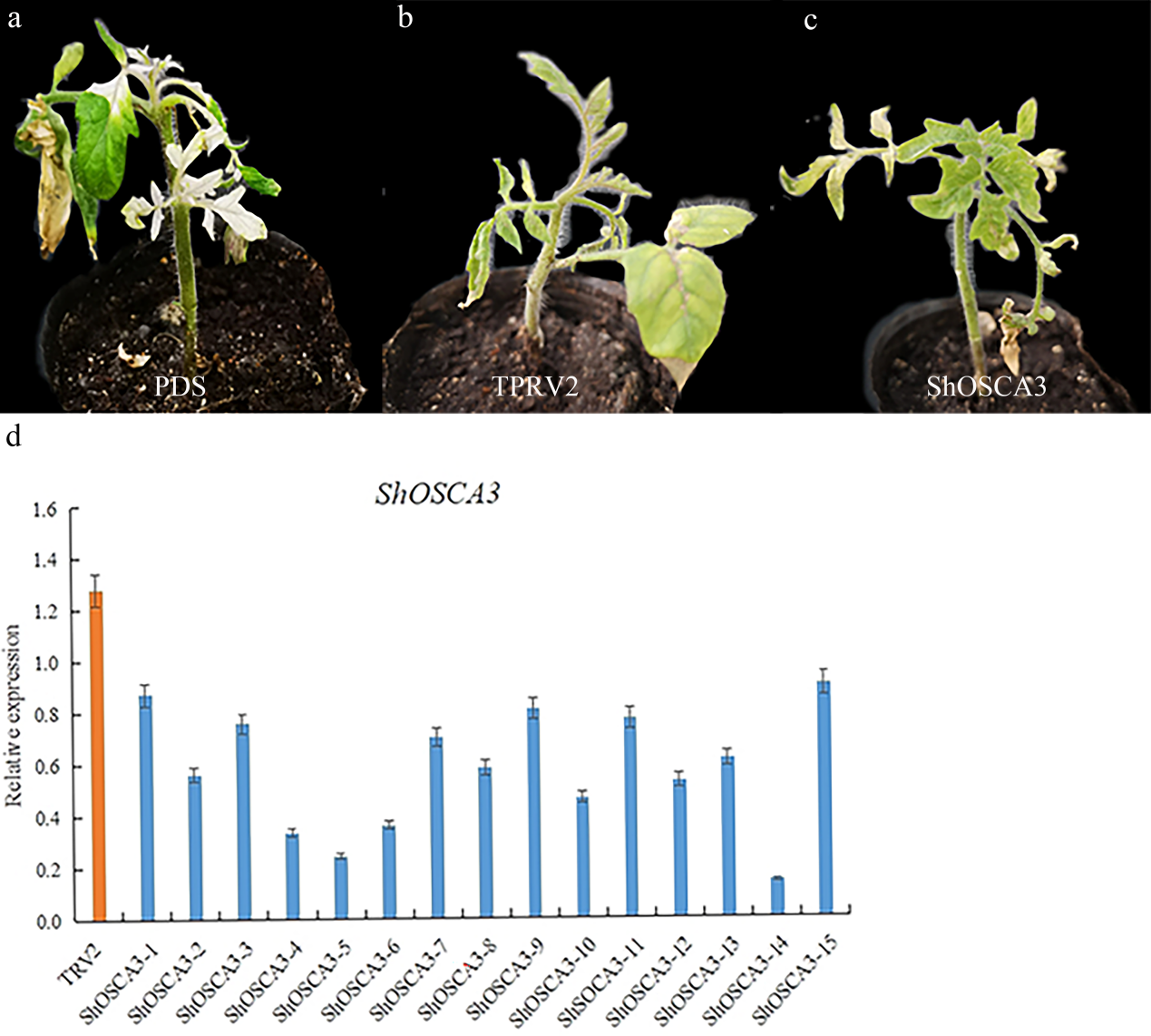


Fig. S2 *ShOSCA3* gene expression in silenced plants. a, b, c, plant phenotypes of *pTRV2-PDS*, *pTRV2*, *pTRV2-ShOSCA3* at 17 days of inoculation of *S. habrochaites*; d, Silencing of *ShOSCA3* confirmed by qRT-PCR.
